# Object files across eye movements: Previous fixations affect the latencies of corrective saccades

**DOI:** 10.3758/s13414-016-1220-6

**Published:** 2016-10-14

**Authors:** Martijn J. Schut, Jasper H. Fabius, Nathan Van der Stoep, Stefan Van der Stigchel

**Affiliations:** Experimental Psychology, Helmholtz Institute, Utrecht University, Heidelberglaan 1, 3584 CS Utrecht, The Netherlands

**Keywords:** Gaze correction, Object correspondence, Visual working memory, Priming, Inhibition of return

## Abstract

One of the factors contributing to a seamless visual experience is object correspondence—that is, the integration of pre- and postsaccadic visual object information into one representation. Previous research had suggested that before the execution of a saccade, a target object is loaded into visual working memory and subsequently is used to locate the target object after the saccade. Until now, studies on object correspondence have not taken previous fixations into account. In the present study, we investigated the influence of previously fixated information on object correspondence. To this end, we adapted a gaze correction paradigm in which a saccade was executed toward either a previously fixated or a novel target. During the saccade, the stimuli were displaced such that the participant’s gaze landed between the target stimulus and a distractor. Participants then executed a corrective saccade to the target. The results indicated that these corrective saccades had lower latencies toward previously fixated than toward nonfixated targets, indicating object-specific facilitation. In two follow-up experiments, we showed that presaccadic spatial and object (surface feature) information can contribute separately to the execution of a corrective saccade, as well as in conjunction. Whereas the execution of a corrective saccade to a previously fixated target object at a previously fixated location is slowed down (i.e., inhibition of return), corrective saccades toward either a previously fixated target object or a previously fixated location are facilitated. We concluded that corrective saccades are executed on the basis of object files rather than of unintegrated feature information.

The human visual system does not build a complete representation of our environment, but instead retains a limited amount of information between eye movements (Irwin & Andrews, 1996; McConkie & Rayner, 1975; Rensink, O’Regan, & Clark, 1997). This information is stored in *transsaccadic memory*, which is thought to be (at least partially) dependent on visual working memory (VWM; Hollingworth & Luck, 2009; Hollingworth, Richard, & Luck, 2008; Irwin, 1991; Luck & Vogel, 2014). However, due to the limited amount of information that can be stored in this memory, selecting the most relevant information is crucial (Irwin, 1992; Luck & Vogel, 1997; Ma, Husain, & Bays, 2014). Voluntary and involuntary orienting of attention guide this selection process (Carrasco, 2011; Hyun, Woodman, & Luck, 2009). Interestingly, visual attention is involuntarily shifted to the location of an intended saccade target just before saccade initiation (Deubel, Schneider, & Bridgeman, 1996; Hoffman & Subramaniam, 1995; Irwin & Gordon, 1998; Van der Stigchel & De Vries, 2015). This presaccadic acquisition of information may be a crucial factor in enabling visual stability (i.e., the sense of a continuous visual world across saccades; McConkie & Currie, 1996). Identifying pre- and postsaccadic information and attributing this information to a specific object facilitates object correspondence across saccades (Hollingworth et al., 2008).

Saccades are somewhat imprecise; therefore, presaccadic acquisition of object information may be particularly useful for distinguishing retinal displacement from object displacement after a saccade. It has been hypothesized that some form of visual search, guided by the surface features in VWM, is used to detect the intended saccade target after the execution of a saccade (Hollingworth et al., 2008; McConkie & Currie, 1996; Richard, Luck, & Hollingworth, 2008). When the intended target is detected, a corrective saccade can be executed to properly foveate the target. To investigate corrective saccades, Hollingworth et al. (2008) designed a gaze correction paradigm. In this paradigm, participants made a saccade toward one of 12 colored disks. During a saccade, the array of disks rotated in such a way that participants had to execute a second (corrective) saccade to land on the target. This corrective saccade process is most likely enabled by the observer presaccadically acquiring the saccade target in memory. The initial saccade target can then be relocated after a displacement during a saccade. In a study using the same corrective-saccade paradigm, participants were tasked to remember unrelated color information for a subsequent memory task in addition to performing the gaze correction task (Hollingworth & Luck, 2009). In this dual-task experiment, it was observed that, when the color information in VWM conflicted with the color of the saccade target, the participants made more erroneous corrective saccades and made corrective saccades with longer latencies. On the basis of these results, the authors concluded that gaze correction targets are acquired in VWM, and therefore are in competition with other VWM contents. These results indicate that the features relevant to a corrective saccade are indeed stored in VWM and can bias corrective-saccade execution. It is currently unknown, however, in what manner previous *attentional* orienting may affect corrective saccades.

Visual attention shares neural substrates with VWM (Mayer et al., 2007) and is thought to underlie the binding of visual features into object representations in VWM (Treisman, 1998; Treisman & Gelade, 1980; Wheeler & Treisman, 2002). Therefore, visual attention allows for future retrieval of these object representations. Currently, it is unclear how the previous attentional deployment may affect corrective-saccade latencies. Previous fixations have been shown to significantly alter saccade latencies in visual search tasks. We hypothesized that, if visual search driven by VWM is indeed a process that precedes the execution of a corrective saccade, then previously attended objects and locations (through fixation) would affect the latencies of corrective saccades. For instance, when searching for a particular target, previously fixated objects and locations are typically less likely to be refixated than are novel objects and locations (Fabius, Schut, & Van der Stigchel, 2016; Mills, Hollingworth, Van der Stigchel, Hoffman, & Dodd, 2011). On the basis of the previous literature, previous fixations could exert two possible influences on corrective saccade latencies.

First, corrective saccades toward a previously fixated target could be executed more slowly because of *inhibition of return* (IOR; see Klein, 2000). IOR is the slowed response (after approximately 200 ms) to previously exogenously attended stimuli (Posner & Cohen, 1984), and is present for both saccadic and manual responses (Reuter-Lorenz, Jha, & Rosenquist, 1996). These effects are tied to objects (instead of to retinal coordinates), as is illustrated by IOR effects at the locations of moving objects (Tas, Dodd, & Hollingworth, 2010; Tipper, Driver, & Weaver, 1991; Tipper, Weaver, Jerreat, & Burak, 1994). In dynamic stimulus displays, an “object” is defined by the previously fixated surface features, such as color or shape, at an updated spatial position. This definition of an object would also be applicable to corrective saccades in the paradigm described by Hollingworth et al. (2008). Furthermore, IOR may slow down decision making in discrimination tasks (Pratt & Abrams, 1999; Terry, Valdes, & Neill, 1994), which may express itself as slowed target acquisition processes in a gaze correction task.

A second possible influence of previous fixations on corrective saccade latencies may be priming effects, in which participants are *faster* to respond (saccadically or manually) to previously attended information (Bichot & Schall, 2002; Godijn & Pratt, 2002; Henderson & Anes, 1994). Indeed, a facilitation of response times by presaccadically acquired information has been observed (Henderson & Anes, 1994). This facilitation in response to presaccadically attended stimuli was present even when the object had shifted location during the saccade. Possibly these results could generalize to the latencies of corrective saccades, as well. In the study by Henderson and Anes, participants used presaccadically acquired information in a passive manner, since that information was not explicitly relevant to the manual-response task. This contrasts with the gaze correction paradigm of Hollingworth and colleagues (2008), in which the presaccadically acquired information is necessary to perform the corrective saccade. Moreover, it has been observed that a target letter is identified more quickly when a similar object (such as another letter) has been fixated previously (Henderson, Pollatsek, & Rayner, 1987), indicating that priming effects may carry over between objects if the objects belong to the same category.

The aim of the present experiment was to examine in what manner corrective saccades to previously fixated objects differ from corrective saccades to nonfixated objects. We expected corrective saccades to a previously fixated target to be either facilitated through object-specific priming or inhibited through IOR. To this end, we adapted the gaze correction paradigm previously described by Hollingworth and colleagues (2008) so that participants had fixated one stimulus in the display prior to executing the gaze correction task. In short, an eye movement to one of six objects was executed before the actual gaze correction task was performed. After refixating, the participant was cued to reorient to either this same object or a different object. During this eye movement the array rotated, and the participant executed a corrective saccade to the object’s updated position.

## Experiment 1

### Method

#### Participants

Twelve participants, of whom ten were female and two male, from ages 19 to 32 years (*M* = 22.5) and from the Utrecht University community, took part in exchange for a monetary compensation of €6 per hour. All participants reported normal or corrected-to-normal vision and were naïve as to the purpose of the study. Written informed consent was obtained from all participants. The study was reviewed and approved by the Faculty Research Ethics Committee of the University of Utrecht.

#### Stimuli and apparatus

In this experiment, six randomly colored circles with a diameter of 1.6° were used as the stimuli. The colors were drawn from a subset of red (12.4 cd/m^2^), green (18.9 cd/m^2^), blue (10.9 cd/m^2^), and magenta (13.6 cd/m^2^). The limitation in this selection was that no circle could be the same color as one of its neighboring circles. The stimuli were presented on a black background (0.34 cd/m^2^). The six colored discs were equidistantly positioned along the circumference of a circle (radius of 4.5°) around a central fixation dot. One circle was cued by expanding its size to 2.1°. A fixation dot (0.6° in diameter, 4.5 cd/m^2^) with a centrally located single black pixel was used as the fixation target and remained on screen throughout the experiment. During each trial, the entire stimulus array rotated by π/6 radians (i.e., 30 deg, or half of the distance between the stimuli) either clockwise or counterclockwise. The rotation event was triggered by gaze positions (see the Procedure section). An outline of a box (2.0° × 2.0°) was drawn around the target stimulus to signal the end of a trial.

The experiment took place in a darkened room. Stimuli were presented on an LG 24MB65PM LCD monitor with a spatial resolution of 1,280 × 800 pixels and a refresh rate of 75 Hz. The size of the screen was 50.8 × 33.9 cm. Participants were seated 70 cm from the monitor with their heads resting on a desk-mounted chin- and headrest. Eye movement data were collected using an EyeLink 1000 (SR Research Ltd., Canada) sampling the left eye at 1000 Hz. Saccades were detected offline with the default values of the EyeLink algorithm for saccade detection.

The experiment was programmed in Python 2.7, using the PyGaze library to connect to the eyetracker and define areas of interest (Dalmaijer et al. 2013). The eyetracker data files were analyzed with Python 2.7, and statistical analyses were performed using R 3.1.3 (Ihaka & Gentleman, 1996).

#### Procedure

Participants performed 460 trials, evenly divided across ten blocks. The eyetracker was calibrated using a standard nine-point calibration procedure prior to starting the experiment. Every trial started with a drift check, which was initiated by the participant by pressing the space bar. The eyetracker was recalibrated whenever the drift was greater than 1.0°. Preceding the experiment, 15 practice trials were performed, which were identical to the experimental trials (see below for a description). The practice trials consisted of five control trials (no rotation during the saccade) and ten experimental trials, in which the target locations were chosen randomly.

**Fig. 1:**
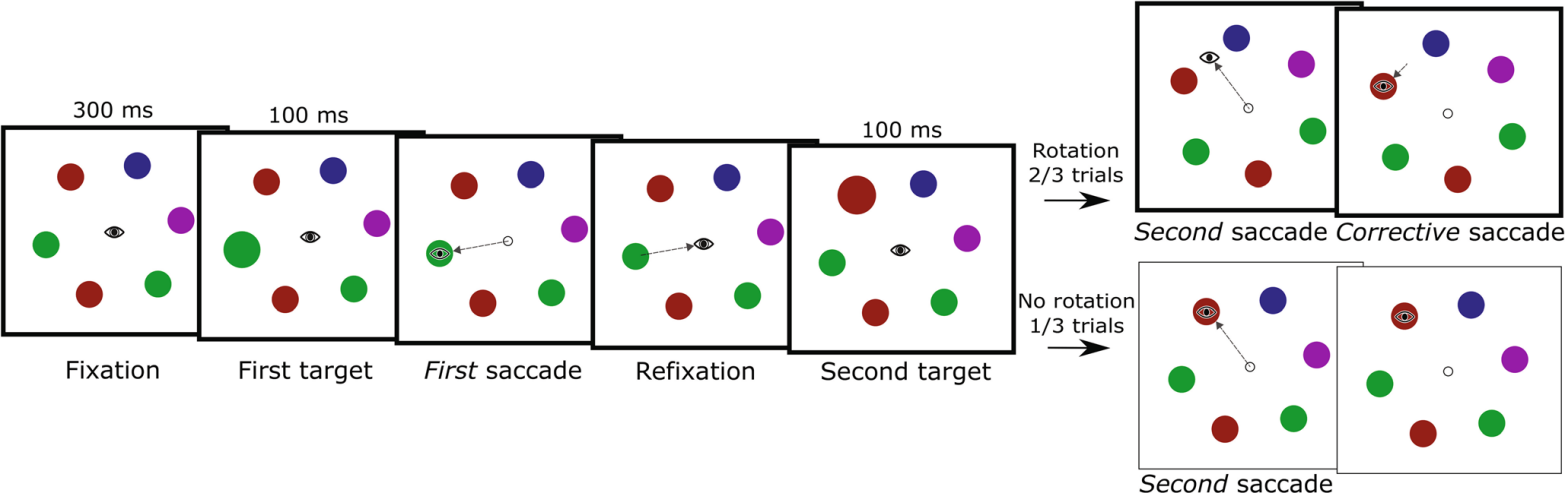
Schematic representation of the procedure used in Experiment 1. The *eye icons* indicate gaze position, and the *dashed lines* indicate saccades. The procedure for the trials in which the array rotated during the second saccade (experimental trials) is outlined in *bold*. Note that the background and fixation stimuli were presented with a contrast opposite to that depicted here

The procedure for the experiment is shown in Fig. 1. After the drift check, a fixation dot (which remained on screen throughout the experiment) was presented for 200 ms. Six colored circles were then displayed in an equidistant circular pattern surrounding the fixation point. To eliminate location-specific effects carrying over between the trials, random orientation offsets (between 1.0 and 35.0 deg) were added to the rotation of the entire array. This orientation offset caused the stimuli never to appear at the exact same location between subsequent trials. One of the six circles was randomly selected as a saccade target, which was visually expanded in size for 100 ms, and subsequently restored to its original size. This object will be referred to as the *first target*. The participant then initiated, as quickly as possible, a saccade to this first target. After fixating the target, gaze was returned to the fixation point. A new randomly chosen colored circle (the *second target*) then expanded for 100 ms, after which it returned to its original size. The participant executed a saccade to this second target. When the participant’s gaze left a 0.4° area of interest surrounding the fixation point, the array of colored circles rotated either clockwise or counterclockwise by 30 deg. Hence, the rotation was such that the participant’s gaze would land between two colored circles, the previously cued circle (the target) and a noncued circle (a distractor). After a delay of 200 ms from saccade offset, a green box (2.0° × 2.0°, line width one pixel) was drawn around the target, signaling the end of the trial and providing feedback on the target identity. By taking the refresh rate of the monitor and other delays (e.g., monitor response time) into account, we retrospectively determined that the onset of the rotation event took place a maximum of 28 ms prior to saccade offset.

Rotations occurred on two thirds of the trials, with no rotations in the remaining third, which prevented participants from making anticipatory corrective saccades. Clockwise rotation and counterclockwise rotation were counterbalanced over the remaining trials and target locations. Combinations of the first and second saccade targets were counterbalanced and appeared equally often, in quasirandom order, between participants. Given these constraints, the first and second targets were the same circle in one out of six trials.

#### Data analysis

**Fig. 2:**
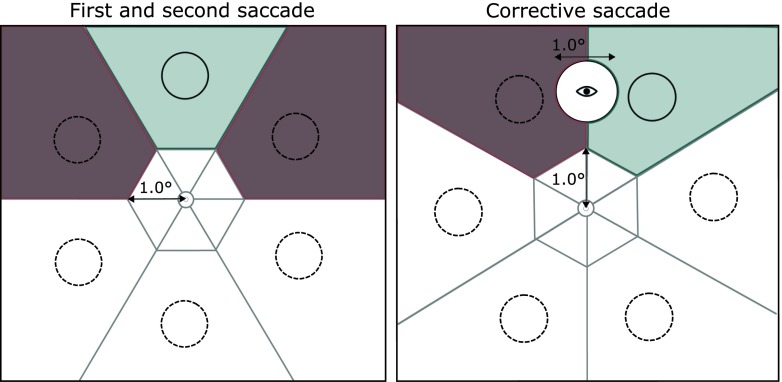
The different regions of interest (ROIs) for the exclusion of saccades for an example trial. The *left panel* shows saccade exclusions for the first and second saccades. The *right panel* shows saccade exclusions for the gaze correction saccades. The eye icon in the right panel indicates initial gaze position after the saccade. The circles with dashed outlines represent distractor stimuli, and the *circle with a black outline* represents the target. The *darker shaded areas* represent the distractor ROIs, and the lighter shaded area the target ROI. A trial was excluded if no samples were present in the lighter shaded area or if samples were present in the darker shaded area

Trials were excluded on the basis of saccade latency and landing position. The saccade latencies for the first and second targets were defined by the difference between the onset of each target cue and the following saccade onset. Saccades toward the first and second targets were excluded when the latency was higher than 1,500 ms (2.1 % of trials). Different regions of interest were used to assess whether the saccade to the first or the second target was executed appropriately (see Fig. 2). The stimulus array was divided using a hexagon extending 1° from fixation. The inner area if the hexagon (up to 1° outward) was indicated as a neutral area. If a participant’s gaze left this area and a sample was detected in the area of interest surrounding a distractor (Fig. 2, left panel), the trial was excluded (3.2 % of trials). Similarly, if no samples were detected in the area of interest around the target, the trial was also excluded (0.1 % of trials). For the corrective saccade, a circular area around the landing position of the *second* saccade (1° diameter) was defined as a neutral area (Fig. 2, right panel). The trial was excluded if the neutral area overlapped with one of the stimulus locations or if the gaze position entered the area of interest around the distractor (5.1 % of trials). We chose to exclude saccades toward distractors because these trials were very infrequent and were unsuited to statistical analyses. We observed a smaller proportion of erroneous saccades than was found with the original paradigm (Hollingworth et al., 2008), which may be due to the reduction of the number of stimuli (from 12 in the original study to six in the present one).

The median saccade latencies for three saccades per trial per participant (the saccades to the first and second targets and the gaze correction) were calculated. Only trials in which a rotation occurred during the saccade to the second target were analyzed. Statistical analyses included paired *t* tests comparing the saccade latencies between conditions in which the same stimulus was cued twice or two different stimuli were cued. Effect sizes are reported as eta squared (*η* ^2^). The gaze correction saccade latency was calculated with regard to the offset of the prior saccade indicated by the saccade detection algorithm. For visualization purposes, grand mean data was plotted with 95 % within-subjects confidence intervals (Cousineau, 2005; Morey, 2008). Furthermore, for the corrective saccades, we show the data per participant, centered around the grand mean, and the slope for each individual (Cousineau, 2005).

## Results

In our analyses we examined two saccades: the saccade to the second cued target (second saccade) and the subsequent corrective saccade that was executed if the array had rotated during the second saccade. We first analyzed the saccade latency of the second saccade in two conditions: when the saccade was executed to either a previously nonfixated circle or a previously fixated circle. We expected the saccades to a previously fixated object to be affected by IOR.

**Fig. 3:**
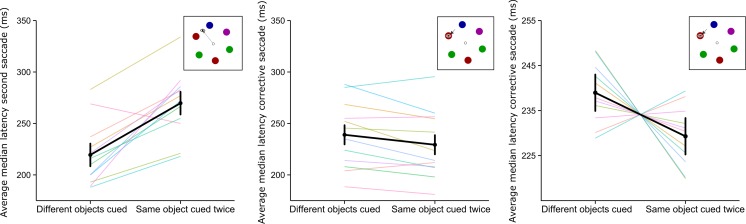
Averages of the median saccade latencies for saccades to objects that were or were not previously fixated. The *bold black lines* show the group averages, with 95 % confidence intervals (in the left and middle panels) and within-subjects 95 % confidence intervals (in the right panel). The *colored lines* indicate the median latencies per participant. The left panel shows the latencies for the second saccade to a target. The *middle and right panels* show saccade latencies for the corrective saccades. The right panel shows the participant data centered around the grand mean, for visualization of within-subjects effects

The latency of the second saccade was significantly higher when a saccade was executed to a previously fixated object (*M* = 269.7 ms, *SD* = 26.7) than when one was executed to a nonfixated object (*M* = 219.4 ms, *SD* = 23.4), *t*(11) = 5.62, *p* < .01, *η* ^2^ = .41 (see Fig. 3, left panel). Analysis of the gaze correction saccades showed that corrective saccades to an object that had previously been fixated but that had shifted during a saccade had a lower latency (*M* = 229.3 ms, *SD* = 26.8) than did corrective saccades to a non-previously-fixated object (*M* = 238.9 ms, *SD* = 25.2), *t*(11) = –2.64, *p* = .02, *η* ^2^ = .04. A visualization of the within-subjects effects is shown in the right panel of Fig. 3, which indicates that nine out of 12 participants seemed to exhibit a lower latency for the corrective saccade when the first and second targets had been the same object. There was no difference in the latencies of the corrective saccades on trials in which the array had rotated clockwise versus counterclockwise, *t*(11) = 0.36, *p* = .73, *η* ^2^ < .01. Together, these analyses suggest that saccades to previously fixated object are initiated more slowly, which is in line with well-studied IOR effects. In contrast, the initiation of subsequent corrective saccades is facilitated.

We further examined whether surface features alone (here, color) could account for the facilitation of corrective saccades to previously fixated items. Consider a trial in which a red disc was cued in the top left of the array and, after refixating the central fixation dot, the participant was instructed to make a saccade to a different red disc in the bottom right of the array. Previous research has shown that congruence between the saccade target information and VWM’s contents may increase saccade latencies in the gaze correction paradigm (Hollingworth & Luck, 2009). In the present study, we observed that saccades to the second target were unaffected by whether or not a different object that had previously been fixated had an identical color to that of the second target, *t*(11) = 0.89, *p* = .38, *η* ^2^ < .02. Similarly, the subsequent corrective saccade did not show an increased or a reduced latency for previously fixated as compared to nonfixated stimuli, *t*(11) = 0.10, *p* = .98, *η* ^2^ < .01. Therefore, surface features of the first cued stimulus do not account for differences in corrective-saccade latencies, even when the surface features were identical to those of the second target object.

### Control analyses

1. The latency of the second saccade affected the latency of the (third) corrective saccade
  It seemed plausible that corrective saccades could be facilitated simply because the preceding saccades had been executed more slowly. To control for whether the facilitation observed was a result of the preceding saccade being slowed, a regression analysis was performed, which included all trials, to estimate the effect of the latency of the second saccade on the latency of the corrective saccade. A *t* test was then used to test whether the mean slope was significantly different from zero. The results are shown in Fig. 4. Overall, in trials in which the saccade to the second target was slow, the corrective saccade was subsequently faster in its onset, *t*(11) = –7.47, *p* < .01. However, comparing the slopes of the trials in which two different objects were cued to the trials in which the same object was cued twice revealed that returning to a previously fixated object still altered this relation between saccade latencies. The relation between slowed saccades to the second target and facilitated corrective saccades was stronger when the same object was cued twice, *t*(11) = –6.01, *p* < .01. Corrective saccades were faster when the prior saccades had been slower if the same object is cued twice (*R* ^2^ = .061), as compared to when two different objects were being cued (*R* ^2^ = .017). Therefore, we concluded that the facilitation of a corrective saccade onset to a previously fixated target was not just due to the inhibition of the onset of the preceding saccade.

**Fig. 4:**
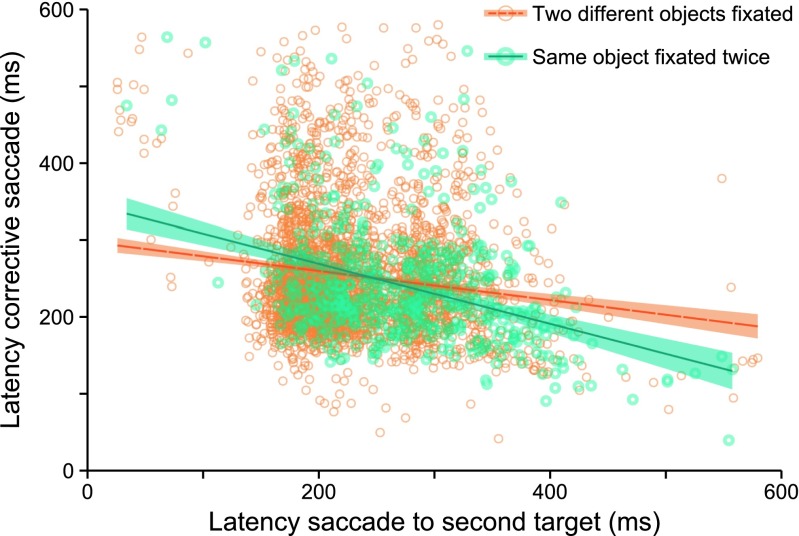
Scatterplot with two regression lines for trials in which two different objects were fixated and trials in which the same object was cued twice. *Shaded areas around the regression lines* represent *SEM*s
2. Latencies of corrective saccades as a function of landing error
  It is also plausible that a corrective saccade might have a lower latency just because it landed close to the corrective-saccade target due to variance in the motor program of executing a saccade. To examine whether the observed facilitation of the onsets of corrective saccades to previously fixated objects can be fully explained by different saccade landing and starting positions, we used linear mixed modeling with maximum likelihood estimation. The statistics reported show a comparison of the Bayesian information criteria (BICs) of one control model and three experimental models. The model with a lower BIC is the model that best explains the variance in the latencies of corrective saccades, where a difference in BICs greater than 10 is generally accepted as strong evidence against the model with a higher BIC (Liddle, 2007; Vrieze, 2012). The control model was constructed with corrective saccade latency as the dependent measure, whether the object had appeared at a previously fixated location as a fixed effect, and a random effect per participant on the intercept. The control model was tested against models that included the second-saccade starting position as a fixed effect, the second-saccade landing position (i.e., the same as the corrective-saccade starting position, barring small gaze shifts) as a fixed effect, or both the saccade landing and starting positions as fixed effects.

The model including the second saccade starting position as a fixed effect did not outperform the control model, *χ* ^*2*^ = 0.30, *p* = .86, nor did the model including a fixed effect for the landing position of the second saccade, *χ* ^*2*^ = 0.60, *p* = .67. Finally, the model including both saccade landing position and saccade starting position did not account for corrective-saccade latencies any better than the control model, *χ* ^*2*^ = 1.17, *p* = .97. Therefore, saccade landing and starting positions did not to seem to contribute to the difference in latencies for corrective saccades between previously fixated and novel objects.

## Discussion

The aim of Experiment 1 was to study the extent to which corrective saccades are influenced by previous fixations. We hypothesized that corrective saccades to previously fixated objects would be affected by either IOR or priming. We found that corrective saccades to a previously fixated object were facilitated. The facilitation in the latencies of corrective saccades is in line with object-specific priming, in which previously attended stimuli are responded to more quickly than unattended stimuli. This finding indicates that the visual system considers previously fixated objects in saccade corrections and that the visual system recognizes such an object as having been previously fixated, although the object was displaced during the saccade. Control analyses excluded alternative explanations such as facilitation as the result of the preceding saccade being slowed by IOR or as the result of different variances in saccade starting and landing positions.

We also analyzed whether the facilitation of the corrective-saccade onset could be accounted for by shared surface features between the objects, since it has been suggested that tracking a particular surface feature may induce attentional effects for objects that share that surface feature (Makovski & Jiang, 2009). However, we did not find a difference in the latencies of corrective saccades to objects that shared versus did not share surface features with the first target. This indicates that the facilitation of a corrective saccade to a previously fixated object is not exclusively explained by surface features shared with a previously fixated object, but seems object-specific. Although two objects may share surface features, the visual system recognizes that these features belong to two different objects, possibly due to contextual cues. These findings provide further evidence that the facilitation of corrective saccade onset is specific to previously fixated objects, rather than to surface feature similarity.

To summarize, we observed that if a target object corresponds to a previously fixated object, the onset of a corrective saccade is facilitated. In Experiment 2, we further investigated whether the representation of an object is updated over time and can induce facilitation of corrective-saccade onset even when its surface features change, or whether maintaining an object correspondence requires that the surface features remain stable—in other words, whether spatial updating alone is enough for the facilitation of corrective saccades. Toward this end, we designed a follow-up experiment that could elucidate whether the stability of surface features is necessary for establishing object correspondence. We hypothesized that a representation of an object is acquired and subsequently can be updated, despite changes in its surface-feature or spatial information—that is, as long as the visual system regards the features as belonging to the same object (Moore, Stephens, & Hein, 2010; Nishida, Watanabe, Kuriki, & Tokimoto, 2007). Additionally, we hypothesized that if the representation of an object can be updated over time, then corrective saccades to a previously fixated object should be facilitated, even when the surface features of the object have changed since fixation of the object. We designed the experiment in such a way that the surface features of the stimuli were ambiguous until the saccade to the first target was executed and the participant had refixated the central fixation point.

## Experiment 2

### Method

#### Participants and procedure

The methods for Experiment 2 were identical to those of Experiment 1, with the exception of the following changes. Twelve participants (eight female, four male; ages 18 to 28 years, *M* = 20.4) participated in the experiment and completed 460 trials each. Two participants who had participated in Experiment 1 also participated in Experiment 2.

**Fig. 5:**
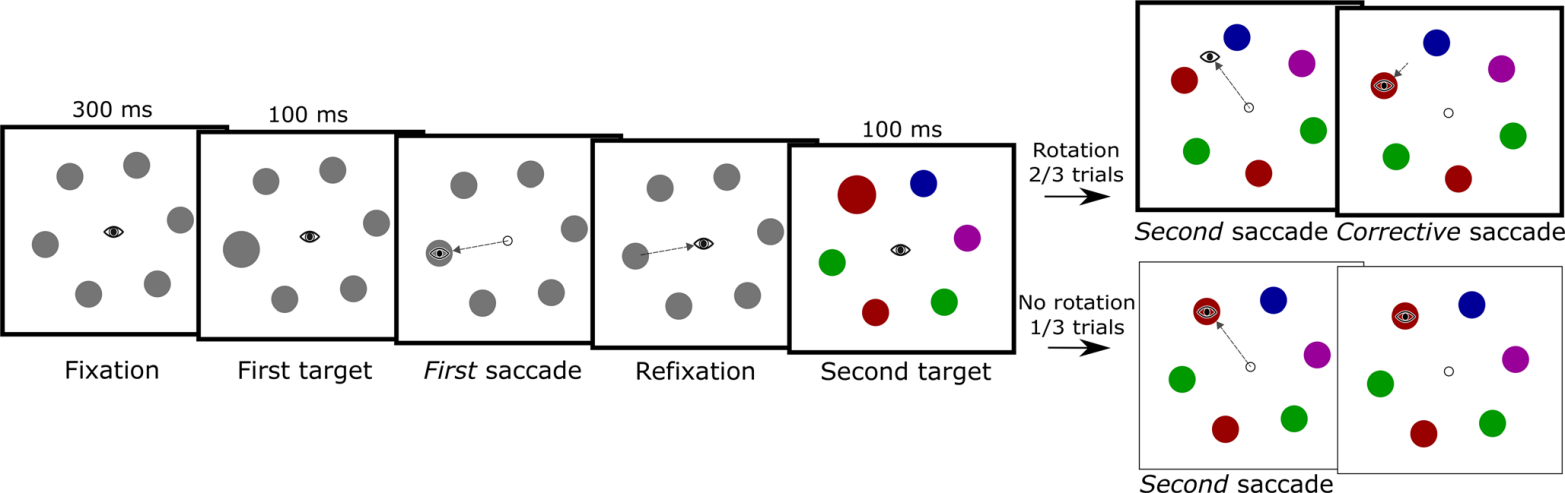
Schematic overview of the paradigm as it was presented in Experiment 2. The eye icons indicate gaze position, and the arrows indicate saccades. The *panels outlined in bold* show the experimental trials. Note that the background and fixation stimuli were presented with a contrast opposite to that depicted here

The crucial manipulation in Experiment 2 was that the color information of the stimuli was not shown before the first saccade, but only after refixating the central point. The stimuli were presented as equiluminant gray circles (10.2 cd/m^2^) until after refixation (Fig. 5). Thus, participants were cued to make a saccade to one of six gray circles and then refixated the central dot. After detection of gaze within 1° of fixation and a subsequent delay of 100 ms, the color of every stimulus changed to one of four colors: red, green, blue, and magenta (as was described in the Method section of Exp. 1). The experiment then resumed as it had in Experiment 1. One of six locations was cued (with a one-in-six chance of the same object being cued twice), and the participant executed a saccade toward this target. In two thirds of the trials, the array rotated either clockwise or counterclockwise during the saccade to the second cued target, causing the participant’s gaze to land between the previously cued item and a distractor. After the saccade landing, the participants performed a corrective saccade to the cued object’s updated position.

## Results

**Fig. 6:**
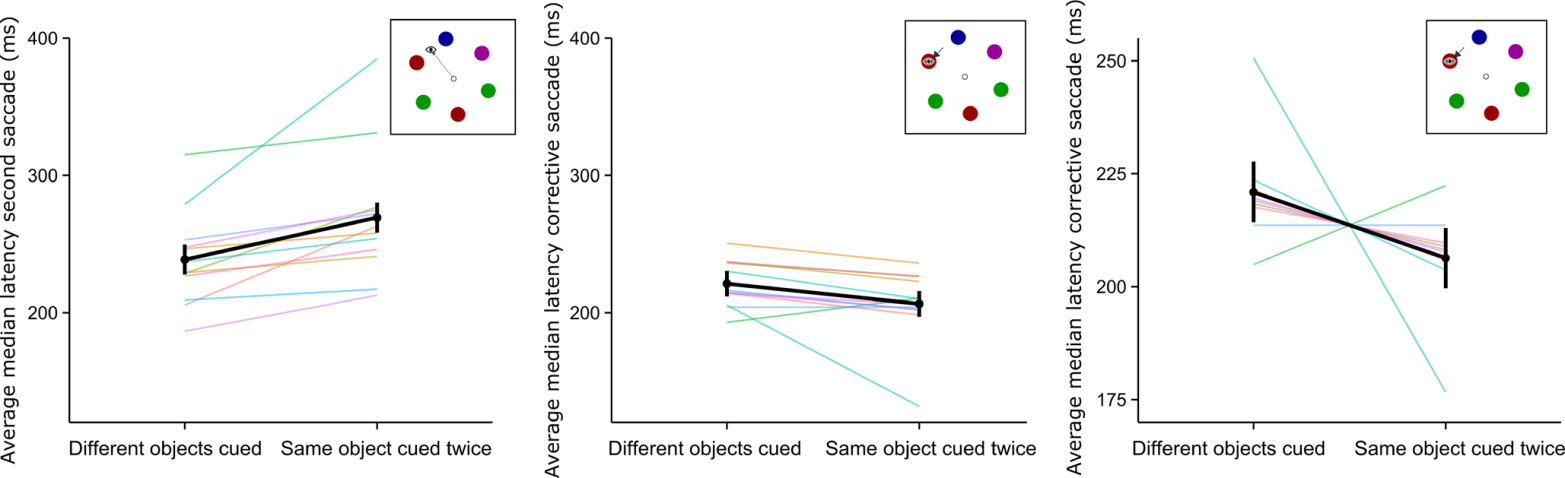
Averages of the median saccade latencies for saccades to objects previously fixated and not previously fixated. The *bold black lines* show the mean data, with 95 % confidence intervals. The *colored lines* show the median latencies per participant. In this experiment, the fixated object changed color between the first and second saccades to the target. The *left panel* shows the latencies for the second saccade to a target. The middle and right panels show saccade latencies for the corrective saccade. The *right panel* shows the participant data centered around the mean and within-subjects confidence intervals

To investigate the contribution of fixated surface features in the facilitation of corrective-saccade onset, we masked the color of the stimuli during fixation, up until the second target was revealed. As is shown in the left panel of Fig. 6, we observed a longer latency for the second saccade when it was executed to a previously fixated object (*M* = 269.3 ms, *SD* = 41.1) rather than a nonfixated object (*M* = 238.6 ms , *SD* = 51.5), *t*(11) = 3.79, *p* < .01, *η* ^2^ = .13, which indicates that IOR was present.

Furthermore, despite the surface features being masked during fixation of the stimulus, we found significant facilitation of the corrective saccade when it was executed to a previously fixated object (*M* = 206.3 ms, *SD* = 31.2), as compared to corrective saccades executed to a nonfixated object (*M* = 221.0 ms, *SD* = 28.6), *t*(11) = –2.39, *p* = .04, *η* ^2^ = .11.

### Control analysis: corrective-saccade latency as a function of second-saccade latency

Across all conditions, we found a significant negative correlation between the latency of the corrective saccade and the latency of the saccade to the second target when it was directed to a novel object, *R* ^2^ = .02. Comparing the baseline correlations for saccades that were executed to previously fixated objects revealed that this relation was strengthened; that is, corrective saccades to previously fixated objects were even quicker when the prior saccades were slowed, *R* ^2^ = .09. The data showed that the slopes (of the correlations between corrective saccades and saccades to the second cued object) were significantly different between targets previously cued and not-previously-cued objects, *t*(11) = 1.61, *p* = .03, *η* ^2^ = .04. Therefore, the alteration in Experiment 2 (as compared to Exp. 1) did not seem to affect the presence of a correlation between these two saccades.

## Discussion

In Experiment 2, we examined whether the constancy of surface features is essential to establishing object correspondence. We hypothesized that the surface features belonging to an object may be updated over time to establish object correspondence. If a surface feature of an object is updated, one might expect that changing color information would not decrease the facilitation of corrective saccades to previously fixated targets. This was indeed what we observed, as well as latencies for first saccades being higher, possibly due to IOR. Importantly, the latencies of corrective saccades to previously fixated objects were still facilitated, as we had observed in Experiment 1, even though irrelevant surface feature information was acquired during fixation of that object. Both Experiments 1 and 2 provide support for the notion that the visual system executes a saccade to an integrated object. That is, whereas corrective saccades to previously fixated features at a fixated location were facilitated, previously fixated features at a different spatial location did not affect corrective-saccade latencies. Furthermore, the results from Experiment 2 add that the properties of this object can be updated when necessary. We concluded that consistency of surface features is not required for establishing object correspondence, and postulated that the process of establishing object correspondence in our paradigm occurs as such: The surface features of the object file are updated during the color shift (e.g., from a gray to a red disk, so that the gray disk information is discarded) before the onset of the second saccade, and the position of the object in the object file is updated after the second-saccade landing (and the target is displaced).

Furthermore, the results showed that the initiation of saccades to a previously fixated object (at a previously fixated location) was again slowed. Inhibition of saccadic return was still present, despite changing surface features after attentional withdrawal. These results are inconsistent with previous research, which had shown that updating the surface features of an object associated with IOR may reduce or eliminate IOR (Tas et al., 2010). On the other hand, our findings are consistent with macaque studies that have shown that IOR is more affected by spatial–temporal incongruencies than by incongruent surface-feature information (Bichot & Schall, 2002). Possibly, the discrepancies between Tas et al.’s and our data could be explained by the target locations changing immediately after refixation in the paradigm of Tas et al., and not changing until during the second saccade in our study.

Our results thus far have shown that the effect of a previous fixation on saccade latencies differs for the second saccade and the corrective saccade. However, in both Experiments 1 and 2 we were unable to conclude that this effect was driven purely by previously fixated locations. The aim of Experiment 3 was to elucidate the effect that previously fixated locations may have on the latency of the second saccade and corrective saccade latency. First, our previous results have shown that corrective saccades executed to previously fixated surface features are facilitated, but the paradigm did not allow the investigation of the effect of previously fixated locations on corrective saccades. The corrective saccades were always executed to a nonfixated location, whereas the second saccade was always performed to either a previously fixated object *and* location, or to neither. Research has shown that IOR can be present at both previously attended locations and objects (Klein, 1988; Klein & MacInnes, 1999; Tipper, Driver, & Weaver, 1991; Tipper, Weaver, Jerreat, & Burak, 1994). Moreover, IOR has been proposed to increase fixation efficiency by lowering the fixation probability of previous fixated locations (Klein, 2000). Considering this, it is not surprising that the second saccade to a previously fixated object and location in Experiments 1 and 2 had a higher latency, since both object and spatial IOR might have affected the saccade latency. In contrast, the subsequent corrective saccade was always executed to a nonfixated location and showed facilitation to previously fixated objects. Currently, it is inconclusive whether the facilitation for the corrective saccade and the inhibition for the second saccade are confounded by an effect of the previously fixated location. We hypothesized that slowed saccade execution for the corrective saccade might also be observed in Experiment 3, but that the second saccade would not show lower latencies when it was executed to previously fixated targets. Second, it is currently unclear whether the higher latency that was observed for the second saccade to a previously fixated than to a nonfixated object is driven by IOR through oculomotor processes (the previously fixated location) or attention (the previously fixated surface features). If the IOR effect for the second saccade were not present after the object had moved, we expected that oculomotor processes related to IOR would underlie the inhibition (Hilchey, Klein, & Satel, 2014; Kingstone & Pratt, 1999). Investigating both of these effects would allow us to assess whether attentional or oculomotor processes underlie corrective-saccade facilitation to previously fixated objects.

Therefore, we altered the design of the paradigm such that we could disentangle the roles of previously fixated *locations* (spatial information) and *objects* (feature information) on both the corrective saccade and the second saccade. In the experimental trials of Experiment 3 the array was rotated twice, once slowly (after the first saccade) and once quickly (during the second saccade). In two thirds of the trials, the layout of the different stimuli *slowly* rotated after refixating the central fixation point, over the course of 500 ms. This rotation was visible to the participant, to facilitate object tracking. In the same trials, the array rotated *quickly* during the saccade to the *second target* (as in Exps. 1 and 2). This setup allowed us to examine corrective saccade latency in three conditions: object congruence, location congruence, and object–location congruence.

*Object congruence* in corrective saccades was determined in the same way described in Experiments 1 and 2. A corrective saccade was executed to a previously fixated object at an updated position. We expected to find results similar to those of our previous experiments, in which corrective saccade latencies were facilitated to previously fixated objects.

In *location congruence*, the target position of a corrective saccade had been previously fixated, but it was occupied by an object that had not been previously fixated. Previous studies have reported location-based IOR in certain spatial-memory tasks that was dissociable from object-specific IOR (Chou & Yeh, 2008; Ludwig, Farrell, Ellis, & Gilchrist, 2009). Additionally, IOR may be elicited by location more than by object identity (Bichot & Schall, 2002), which seems to be supported by neurophysiological studies. The superior colliculus is critically involved in executing saccades and (disengaging) spatial attention (Dash, Yan, Wang, & Crawford, 2015; Ferreira, Araujo, Matsumoto, Ono, & Nishijo, 2015; Sapir, Soroker, Berger, & Henik, 1999; Wurtz & Goldberg, 1972). As such, activity in the superior colliculus has been shown to reflect IOR (Dorris, Klein, Everling, & Munoz, 2002; Fecteau, Bell, & Munoz, 2004), which is supported by lesion studies showing that IOR is not generated in patients with a lesioned superior colliculus (Sapir et al., 1999). In location-congruent trials, a corrective saccade would be generated to a previously fixated location, which we hypothesized would be slowed by the previous attending of the target location. The alternative is that corrective saccades to previously fixated locations would be facilitated at onset, because IOR has been implicated as a novelty-seeking mechanism (Posner & Cohen, 1984; Taylor & Klein, 1998; Wang & Klein, 2010). The novelty, which might elicit facilitation (Courchesne, Hillyard, & Galambos, 1975), in location congruence was that a new object (not previously fixated) was occupying a previously fixated location. Furthermore, studies have shown that IOR is very much task-dependent, and location-specific cueing may occur instead, depending on the task demands (Dodd, van der Stigchel, & Hollingworth, 2009).

Finally, *object*–*location congruence* was a condition in which the corrective saccade would be executed to a previously fixated object at a previously fixated location. As we mentioned previously, we hypothesized that this might induce inhibition of saccadic return, because this saccade would be executed to a location and object that had previously been fixated, similar how to the second saccade was slowed in Experiments 1 and 2 when it was executed to a previously fixated location and object. When the corrective-saccade target was congruent in terms of both the previously fixated object features and location, this should provide evidence to the visual system that this location had previously been attended, to elicit the object-specific IOR described by Tipper et al. (1994). These results will provide insight into the interplay between both object- and location-specific information by the visual system to establish object correspondence.

## Experiment 3

### Method

#### Participants and procedure

In Experiment 3, 16 participants between 18 and 27 years of age (*M* = 20.1) completed 648 trials each. The procedure was as follows. A trial was the same as in Experiment 1 up until refixation of the central fixation point after the first saccade. To reiterate, participants were presented with six colored circles. One of these circles was cued (first saccade target). Participants executed a saccade to this object, after which they refixated the central point. The experimental procedure then changed with regard to Experiment 1. In two thirds of the trials, the array rotated visibly over the course of roughly 500 ms (37 frames; *slow* rotation) while the participants fixated the central point. This slow rotation could occur either clockwise or counterclockwise, which were counterbalanced to occur equally often per participant. During the slow rotation, the stimulus array was rotated exactly 30 deg. After the rotation had concluded, one of the six objects was cued as being the second saccade target. In one out of six trials, the same object was cued twice, albeit at a different location between the cues, due to the slow rotation. Once a saccade was executed, the stimuli rotated another 30 deg either clockwise or counterclockwise (*fast* rotation). The fast rotation, as in Experiments 1 and 2, occurred during one refresh cycle of the monitor. Finally, the participants executed a corrective saccade to the target’s updated position. Rotating the array twice meant that the stimuli could rotate either back into a position that was congruent to when the object had been fixated initially or to a position that had been occupied by a different object during the saccade to the first target.

Trials in which no slow rotation occurred were added, to prevent participants from anticipating the slow rotation. In trials without slow rotation, the cue to the second target was delayed by 37 frames (approximately 500 ms), thus keeping the time frames identical between both types of trials. Only trials in which the array rotated twice were used to investigate corrective saccade latencies and will be referred to as the *experimental trials*.

**Fig. 7:**
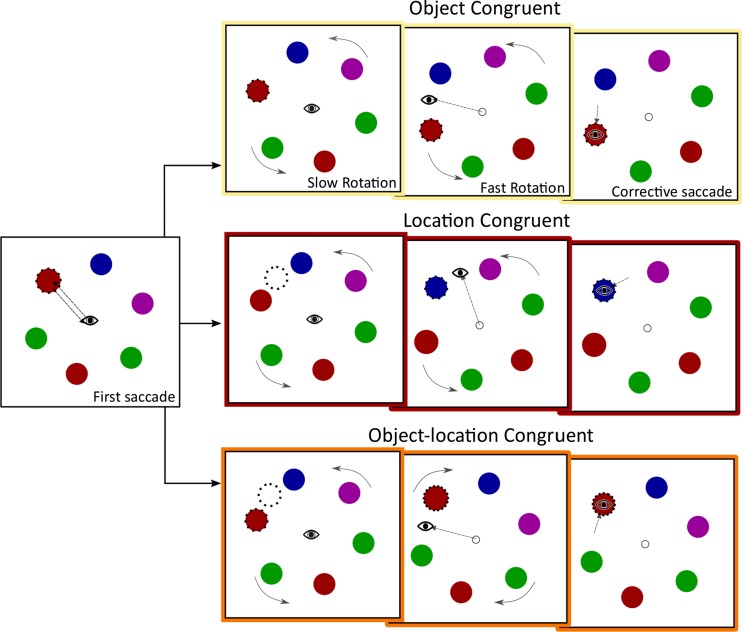
Three example trials in Experiment 3. The first panel shows the experiment up to the rotation during refixation. For illustrative purposes, a *dotted line* has been drawn around the previously fixated object (*top panels*), location (*middle panels*), or object and location (*bottom panels*). The *top panels* show a situation in which a previously fixated object is at a not-previously-fixated location (object-congruent trials). The *middle panels* show a situation in which a previously fixated location contains a not-previously-fixated object (location-congruent trials). The *lower panels* show the trials in which the corrective saccade was executed to the same object and location that had previously been fixated (object–location-congruent trials)

As we mentioned above, this procedure allowed us to examine a number of situations that may affect corrective-saccade latencies, of which three example experimental trials are shown in Fig. 7. We were interested in studying these particular conditions for corrective saccades: object-congruent trials, in which only the object had been previously fixated; location-congruent trials, in which only the location had previously been fixated, with a novel object occupying this location; and object–location-congruent trials, in which the corrective saccade was executed to both a location and object that had previously been fixated.

To clarify, object-congruent trials (i.e., a previously fixated object at an updated position, as is shown in the top panels of Fig. 7) would be induced by executing the first saccade to one of six objects. In the example shown in Fig. 7, the slow rotation occurred counterclockwise. The previously fixated object was cued a second time, and during the saccade to this object the fast rotation occurred counterclockwise once more, causing the object to appear at a nonfixated location. After the second-saccade landing, the participant therefore executed a corrective saccade to a previously cued object. Object-congruent trials could also occur by cueing the same object twice and rotating the array clockwise during both the slow and fast rotations. In object-congruent trials, the corrective saccade was therefore executed to a fixated object that was rotated to the position of a nonfixated neighboring object.

**Fig. 8:**
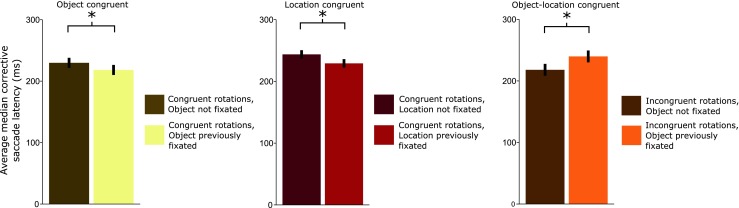
Average median latencies for corrective saccades. The *left panel shows* latencies for corrective saccades to an object that had previously been fixated versus a nonfixated object; the middle panel shows latencies for corrective saccades to a previously fixated location occupied by a nonfixated object versus to a previously nonfixated location. The *right panel* shows latencies for previously fixated objects at a previously fixated location versus trials in which neither the object nor the location was fixated. *Congruency* refers to slow and fast rotation occurring in the same direction—for example, both clockwise. *Error bars* represent 95 % confidence intervals

**Fig. 9:**
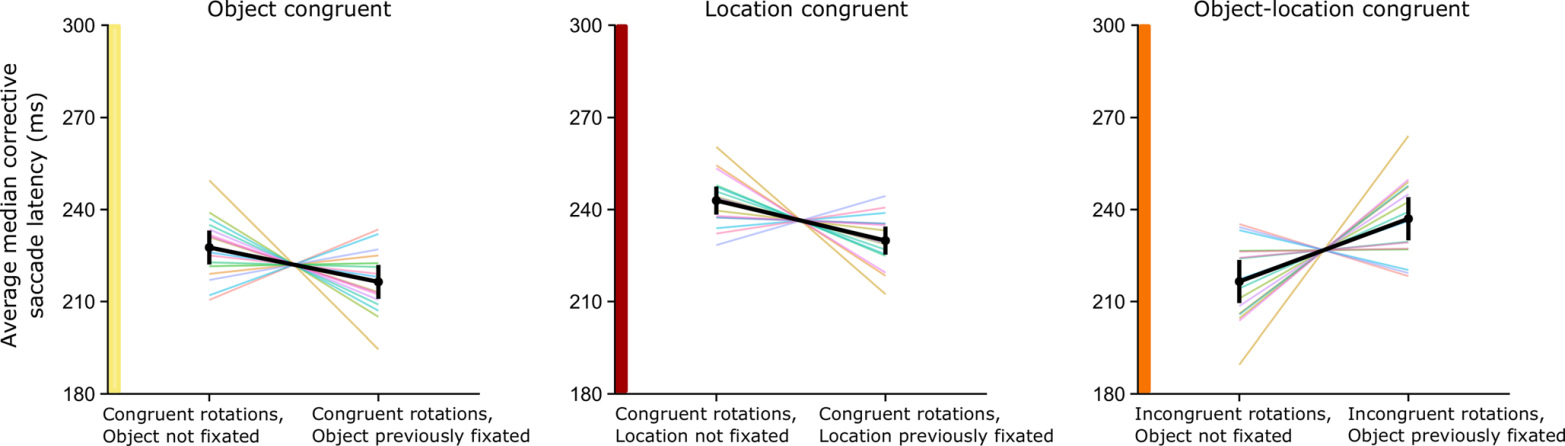
Within-subjects effects of previous fixations on median saccade latencies per participant, centered around the grand mean. The calculated grand mean is shown as a black line with 95 % within-subjects confidence intervals. The left panel shows latencies for corrective saccades to a previously fixated object at a novel location; the middle panel shows latencies for corrective saccade to a previously fixated location only; and the right panel shows latencies for a previously fixated object and location

In the location-congruent trials (indicated by the middle panels of Figs. 7, 8 and 9), in which only the location was previously fixated, we cued one of six objects. Again, both the fast and slow rotations were congruent (e.g., both clockwise), but two neighboring items were cued. For example, the first saccade was executed toward the top-left item, and after a counterclockwise slow rotation, the top item (one item to the right) was cued. During the saccade to this target, the array rotated counterclockwise again. This rotation led to a nonfixated object occupying a location that had previously been fixated.

Object–location-congruent trials (bottom panels in Fig. 7) occurred if the same object was cued twice. In these trials, the slow and fast rotations occurred in opposite directions. In the example shown in Fig. 7, the slow rotation occurred counterclockwise, followed by a clockwise rotation during the second saccade. Therefore, the corrective saccade was executed to both the same object and location that had previously been fixated.

These three types of trials (object congruent, location congruent, and object–location congruent) were analyzed separately. In all analyses, the three trial conditions were compared to situations in which the slow and fast rotations occurred in the same direction, but with different items cued between the first and second saccades.

## Results

We analyzed three types of corrective-saccade trials: object-congruent, location-congruent, and object–location-congruent trials. The main results are shown in Fig. 8, and individual participant data are shown in Fig. 9.

For object-congruent trials, we found significant facilitation, *t*(15) = –2.22, *p* = .04, *η* ^2^ = .02, for corrective saccades to a previously cued object at a novel location (*M* = 216.4 ms, *SD* = 49.3) as compared to a non-previously-cued object at a novel location (*M* = 227.6 ms, *SD* = 49.0); see the left panel of Fig. 8. For location-congruent trials, in which a repeated location was fixated but a novel object occupied it (*M* = 235.5 ms, *SD* = 41.0), we found significant facilitation, *t*(15) = –3.04, *p* < .01, *η* ^2^ = .04, relative to a non-previously-fixated location with a novel object (*M* = 244.3 ms, *SD* = 46.7). Finally, the analysis for object–location-congruent trials indicated that corrective saccades were slowed significantly, *t*(15) = 3.09, *p* < .01, *η* ^2^ = .10, when they were directed to a previously fixated object and location (*M* = 237.0 ms, *SD* = 54.4), as compared to corrective saccades to a non-previously-fixated object and location (*M* = 216.5, *SD* = 44.8), shown in the right panel of Fig. 8.

Finally, we examined the effects of the slow rotation on latencies of the second saccade. In our previous experiments, corrective-saccade latencies were correlated with the prior saccade latencies. This correlation had been stronger when saccades were executed to both a previously fixated object and location in the previous experiments. In Experiment 3, the slow rotation caused the target of the second saccade to be in a different location than when it had previously been fixated, which might elicit either object-specific facilitation or inhibition. Therefore, we investigated how the latencies of second saccades were affected by the slow rotation we introduced, since the absence of object-specific inhibition for the second saccade might affect the latencies of the corrective saccade. To analyze the effects of the slow rotation on second-saccade latencies, we performed a repeated measured analysis of variance. The dependent variable was the median saccade latency of the second saccade, with slow rotation (clockwise, counterclockwise, or no rotation) and stimulus fixated or not fixated previously as independent variables. We found main effects of both previously fixating the stimulus, *F*(1, 15) = 42.97, *p* < .01, *η* ^2^ = .13, and the direction of slow rotation, *F*(2, 30) = 11.78, *p* < .01, *η* ^2^ = .10, on the latencies of the second saccade, and an interaction between these two factors, *F*(2, 30) = 14.50, *p* < .01, *η* ^2^ = .03. We examined the interaction effect in further detail by using Holm–Bonferroni-corrected *t* tests for post-hoc analyses.

**Fig. 10:**
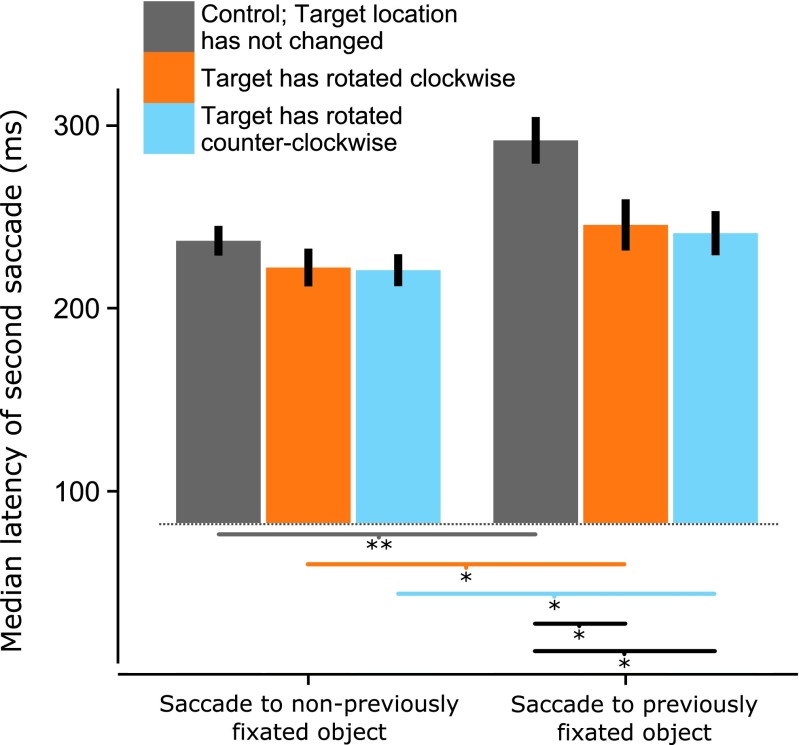
Latencies for saccades to a second cued target. The *colored bars* show trials in which no slow rotation was present and trials in which slow rotation was present prior to executing the saccade. The *bars on the left* represent the condition in which a saccade to a not-previously-fixated object was executed, and the bars on the right show saccade latencies to a previously fixated object, which had an updated position for some of the bars. The lines underneath the bars show significant post-hoc comparisons, and error bars show 95 % confidence intervals. ^*^ *p* < .05, ^**^ *p* < .01

Significant differences are indicated by the asterisks in Fig. 10. First, in trials in which no slow rotation was present (similar to Exp. 1), we found a significant increase in second-saccade latencies for saccades to previously fixated objects relative to not-previously-fixated objects, *t*(15) = –6.13, *p* < .001. We also found a significant increase in second-saccade latencies when a saccade was executed to a previously fixated object that had rotated visibly in both the clockwise and counterclockwise rotation conditions, *t*(15) = –4.90, *p* = .01, and *t*(15) = –4.03, *p* = .01. Comparing saccades executed to previously fixated objects revealed that the latency of the second saccade in the no-slow-rotation condition was significantly higher than those in either the clockwise, *t*(15) = –1.88, *p* = .04, or the counterclockwise, *t*(15) = –2.26, *p* = .02, condition. Other post-hoc comparisons yielded nonsignificant results. We concluded that the saccade latency was higher to a previously fixated target, even when the target had moved since fixation, although incongruent spatial information might lessen the magnitude of this inhibition.

## Discussion

In Experiments 1 and 2, we observed that corrective saccades to previously fixated objects were facilitated in terms of saccade onset latencies. However, these corrective saccades were always performed to a location that had not previously been fixated. In Experiment 3, we investigated corrective saccade latencies to both previously fixated objects and previously fixated locations.

Interestingly, when either the target object or the target location had previously been fixated, corrective saccades were facilitated. However, we found inhibition of corrective saccade onset when both the same object *and* location had previously been fixated. In Experiments 1 and 2, corrective saccades had been executed faster to previously fixated objects (at nonfixated locations) than to nonfixated objects. In contrast, in Experiment 3 the second saccade was executed more slowly to either a previously fixated object or a previously fixated object and location than to a nonfixated object at a nonfixated location. Importantly, the second saccade and the corrective saccade were differentially affected by the previously fixated locations. This could be taken to suggest that the second saccade and the corrective saccade were differentially affected by attention and oculomotor programming. Moreover, our results indicate that saccades to previously fixated stimuli lead to an increase in subsequent corrective-saccade latencies (i.e., slower saccades) only when there is both object *and* location congruency. These findings are perhaps explained by some lingering of IOR at the previously fixated location, which is only activated in the absence of novel information (i.e., a new object or location).

Our results indicate that corrective saccades were initiated faster when they were executed to a previously fixated object or location. These results imply that for saccade execution, the visual system independently weighs the previously fixated surface features (as object correspondence operations) and previously inspected locations.

## General discussion

Previous research has indicated that both object-specific IOR (Tipper et al., 1991) and object-specific priming (Henderson et al., 1987) can affect the processing of previously viewed stimuli. Here, we conducted a series of gaze correction experiments to investigate how previous fixations may alter corrective-saccade onset. More specifically, we investigated whether surface features, such as color, and location information would independently affect corrective saccades. To this end, we cued participants to fixate one of the objects in an array prior to performing the corrective-saccade task. Our study shows that object-specific priming and IOR may affect corrective-saccade latencies differently under different circumstances. Corrective saccades were *faster* when they had to be executed to a new object at a previously fixated location, or when a previously fixated object had moved to a new location. In addition, we observed a *slowing* of corrective saccades to previously fixated objects when they were positioned at the same location at which the object had initially been inspected (similar to IOR). The mechanisms that underlie these effects are currently unclear. However, the present results suggest that performing a corrective-saccade task involves processes similar to performing a visual search task, given that the facilitatory and inhibitory effects occur under highly similar conditions (Dodd et al., 2009). These observations could be taken to suggest that the visual system compares remembered features to the current sensory input and initiates a saccade when surface-feature or spatial information is detected that is incongruent with memory’s content.

Recent studies on transsaccadic integration of object features has shown that pre-saccadically-acquired peripheral information and subsequent foveal information are integrated after a saccade has been completed (Herwig 2015; Oostwoud Wijdenes et al., 2015; Wolf & Schütz, 2015). These studies showed that the visual system weighs both peripheral and foveal information and creates one percept. Even more, integration is only present when the visual system considers the pre- and postsaccadic information to belong to the same object (Ganmor, Landy, & Simoncelli, 2015; Wolf & Schütz, 2015). The phenomenon of transsaccadic integration implies that VWM representations are not stable over time, but rather are constantly updated by new visual information. Our study extends this hypothesis by showing that object correspondence, despite a mismatch of location, is not driven by template matching of previously viewed features (e.g., matching any red object to another red object to establish object correspondence). We suggest that the visual system constantly is either updating object information and integrating remembered and new information into one object representation or replacing (i.e., disregarding the previous) the information present in VWM between saccades, on the basis of the task demands.

The current literature seems to agree with the idea that corrective saccades are guided by two kinds of input: visual and motor. To illustrate the differences, we would note that corrective saccades guided by memory contents (*visual* corrective saccades; 200 to 300 ms) are generally slower than corrective saccades in response to variance in motor execution (*motor* corrective saccades; latencies of 50 to 200 ms; Becker, 1976). Motor corrective saccades are executed when gaze after a saccade has not reached the intended target. These motor corrective saccades are likely to be executed more quickly than visual corrective saccades, since the motor program that has been executed (saccadic corollary discharge) is available to the visual system before the saccade has reached its landing position (Bridgeman, 1995; Collins, Rolfs, Deubel, & Cavanagh, 2009). The efference copy of the corollary discharge, which (ultimately) allows a motor corrective saccade to be executed after the previous saccade has missed its target, has been shown to contribute to perceived visual stability (Cavanaugh, Berman, Joiner, & Wurtz, 2016). Yet the implications of visual corrective saccades for visual stability are not as clearly defined. Both motor and visual corrective saccades have been linked to each other by studies that have shown that motor corrective saccades benefit in terms of accuracy from the availability of visual information upon landing (Tian, Ying, & Zee, 2013). Furthermore, visual corrective saccades are difficult to suppress and are likely to be executed even when they are not task-relevant (Exp. 4 in Hollingworth & Luck, 2009). In our study, participants could not use their copy of the corollary discharge to successfully complete the corrective-saccade task (because the displacement of stimuli was artificial), indicating that object correspondence (as indicated by object-specific facilitation of saccade onsets to previously fixated objects) can be established by visual information alone. We interpret these results as evidence that both the corollary discharge signal and visual information can guide corrective saccades, depending on the availability of information before saccade onset.

In conclusion, object correspondence is initiated by congruency between remembered surface features and spatial information and promotes visual continuity. Our study adds that previously attended visual information can significantly affect the time course of establishing object correspondences across saccades, as indicated by changes in saccade latencies.
